# In Silico Investigation of Phytochemicals from Djiboutian Plants Targeting Sulfate and Phosphate Transporters Involved in Dichromate Uptake

**DOI:** 10.3390/ph19071000

**Published:** 2026-06-28

**Authors:** Fatouma Mohamed Abdoul-Latif, Oussama Abchir, Abdirahman Elmi, Lamiae El Bouamri, Talal Mohamed, Imane Yamari, Ricardo Gil-Ortiz, Pannaga Pavan Jutur, Samir Chtita

**Affiliations:** 1 Medicinal Research Institute, Center for Research and Study of Djibouti, Djibouti 486, Djibouti; 2 Laboratory of Analytical and Molecular Chemistry, Faculty of Sciences Ben M’Sick, Hassan II University of Casablanca, Casablanca 7955, Morocco; 3 Independent Researcher, E-46022 Valencia, Spain; 4 International Centre for Genetic Engineering and Bio-Technology (ICGEB), Aruna Asaf Ali Marg, New Delhi 110067, India

**Keywords:** hexavalent chromium (Cr(VI)), sulfate transporter, phosphate transporter, phytochemicals, plant–pollutant interaction, molecular docking, molecular dynamics

## Abstract

**Background/Objectives:** Chromium contamination represents a major environmental challenge due to its detrimental effects on plant growth and agricultural productivity. Since dichromate uptake in plants occurs mainly through sulfate and phosphate transporters, identifying natural compounds capable of competitively inhibiting these transport pathways may provide an eco-friendly strategy for reducing chromium accumulation. This study aimed to investigate the inhibitory potential of phytochemicals from Djiboutian medicinal plants against sulfate and phosphate transporters using an integrated computational approach. **Methods:** 49 phytochemicals identified by GC–MS from ten Djiboutian medicinal plants were screened against the sulfate transporter (7LHV) and phosphate transporter (7SP5) using molecular docking. Binding interactions were compared with sulfate, phosphate, and dichromate ions to evaluate potential competitive inhibition. The most promising compounds were further assessed through ADMET prediction and 100 ns molecular dynamics simulations to evaluate their pharmacokinetic properties and complex stability. **Results:** Molecular docking revealed binding energies ranging from −7.04 to −2.91 kcal/mol for 7LHV and from −6.50 to −0.62 kcal/mol for 7SP5, indicating variable binding affinities among the screened phytochemicals. Several compounds exhibited favorable interactions with key amino acid residues involved in anion transport, suggesting their potential to compete with dichromate uptake. ADMET analysis identified multiple compounds with favorable toxicity and drug-likeness profiles. Among them, cyclohexanepropanoic acid from Aloe djiboutiensis demonstrated the strongest binding affinity toward both transporters. Molecular dynamics simulations confirmed the structural stability of the protein–ligand complexes throughout the 100 ns simulation. **Conclusions:** This study identifies naturally occurring phytochemicals, particularly cyclohexanepropanoic acid, as promising competitive inhibitors of dichromate transport in plants. These findings provide a theoretical foundation for developing sustainable phytochemical-based strategies to mitigate chromium accumulation in crops and support future experimental validation.

## 1. Introduction

Chromium toxicity caused by hexavalent chromium (Cr(VI)) has become a major environmental and agricultural concern due to the extensive release of chromium-containing industrial effluents from tanning, electroplating, textile, mining, and metallurgical activities [1]. Among chromium species, Cr(VI) is considered the most toxic form because of its high solubility, mobility, and oxidative potential, enabling its rapid dissemination in soil and water ecosystems [2]. Once introduced into agricultural environments, Cr(VI) can be readily absorbed by plants and accumulated in edible tissues, thereby threatening crop productivity, food security, and human health through trophic transfer [2].

In plant systems, dichromate and chromate oxyanions exploit existing anion transport pathways due to their structural similarity to essential nutrients such as sulfate and phosphate ions [3,4]. Previous studies demonstrated that sulfate transporters play a major role in Cr(VI) uptake, while phosphate transporters may also contribute to the internalization of toxic oxyanions [5,6]. This competitive transport mechanism results in oxidative stress, membrane damage, metabolic perturbations, and inhibition of plant growth and photosynthetic activity [3]. Consequently, limiting chromium uptake at the transporter level represents a promising strategy for reducing chromium accumulation and toxicity in plants [5,6].

Natural products derived from medicinal plants constitute an important source of structurally diverse bioactive molecules with potential environmental and pharmacological applications. In recent years, phytochemicals have attracted considerable attention owing to their antioxidant, metal-chelating, and protective properties [7]. Advances in computational chemistry have further facilitated the exploration of plant-derived compounds as potential modulators of biological targets. Molecular docking, ADMET prediction, and molecular dynamics simulations are now widely used to predict ligand–protein interactions, evaluate binding stability, and identify promising inhibitory compounds prior to experimental validation [8,9,10,11].

The medicinal plants investigated in the present work are native to Djibouti and remain poorly explored regarding their phytochemical composition and biological potential. GC–MS analysis of the plant extracts enabled the identification of a diverse library of secondary metabolites belonging to different chemical classes, including alcohols, terpenoids, phenolics, ketones, and nitrogen-containing compounds [12]. Considering the structural diversity and potential bioactivity of these metabolites, it is hypothesized that some phytochemicals may competitively bind to sulfate and phosphate transporters and interfere with dichromate transport mechanisms.

The present study was undertaken to investigate the volatile phytochemical constituents of ten plant species collected in Djibouti and to explore their potential role in mitigating dichromate uptake through the inhibition of sulfate and phosphate transporters. Although many volatile compounds are widely distributed among plant species, their occurrence and relative abundance may vary according to environmental and geographical conditions. Given the limited phytochemical information available for Djiboutian flora, the characterization of these species contributes to expanding current knowledge of their chemical composition. Furthermore, the identified phytochemicals were subjected to in silico analyses to evaluate their potential interactions with transport proteins implicated in dichromate uptake. Thus, the significance of this study lies not only in the phytochemical profiling of underexplored plant species but also in the identification of candidate natural compounds that may contribute to reducing chromium accumulation in plants.

Therefore, the objective of this study was to investigate, through an integrated in silico approach, the binding mode of phytochemical compounds identified from ten Djiboutian medicinal plants with the sulfate transporter 7LHV and the phosphate transporter 7SP5. Molecular docking simulations were performed to evaluate binding affinities and interaction modes, followed by ADMET analysis to assess pharmacokinetic and toxicity properties. The most promising compounds were subsequently subjected to molecular dynamics simulations in order to evaluate the stability and persistence of ligand–receptor interactions over time. This work provides new insights into the potential application of plant-derived natural compounds as eco-friendly competitive inhibitors capable of reducing chromium uptake and accumulation in plants.

## 2. Results and Discussion

### 2.1. GC–MS Profiling of the Studied Medicinal Plants

The GC-MS profiling analysis of the ten Djiboutian medicinal plant extracts revealed a chemically diverse composition characterized by the presence of numerous volatile and semi-volatile secondary metabolites. The extraction yields varied considerably among the investigated plants, ranging from 2% for *Aloe ericahenriettae* to 16% for *Jasminum floribundum*, reflecting differences in metabolite abundance and chemical composition (Table 1). Overall, 49 phytochemical compounds were identified from the analyzed extracts. The detected compounds belonged to several important phytochemical families, including terpenoids, alcohols, ketones, hydrocarbons, esters, phenolic derivatives, and nitrogen-containing metabolites. Such chemical diversity suggests the presence of compounds with potentially significant biological and environmental activities (Table S1).

The GC–MS results provided the molecular basis for the subsequent computational investigations. The identified phytochemicals were therefore selected as candidate ligands for evaluating their ability to competitively inhibit dichromate uptake through sulfate and phosphate transport systems. The observed structural diversity among the detected metabolites may explain the variation in docking scores and interaction profiles obtained during molecular modeling analyses.

### 2.2. Molecular Docking Results

Molecular docking of all 49 compounds was performed within the binding site of the sulfate transporter, and both the binding energy (ΔG) and inhibition constant (Ki) were calculated for each docked ligand. The results, summarized in Table 2, showed that the binding energies of the phytochemical compounds ranged from −2.91 to −7.04 kcal/mol, compared to −3.77 kcal/mol for sulfate and −9.59 kcal/mol for the dichromate ion. Correspondingly, the calculated Ki values for the compounds varied between 6.91 μM and 7.3 mM, while the reference ligands exhibited Ki values of 1.71 mM (sulfate) and 92.64 nM (dichromate). These results indicate a range of predicted inhibitory potentials, highlighting several phytochemicals with promising competitive interactions relative to the native ligands.

The phosphate transporter exhibited binding energies ranging from −6.50 to −0.62 kcal/mol, with inhibition constant (Ki) values varying between 15.71 µM and 353.51 mM. In comparison, the reference dichromate ion showed a binding energy of −10.13 kcal/mol and a Ki value of 37.58 nM, whereas the sulfate ion displayed a binding energy of −2.13 kcal/mol with a Ki of 27.5 mM. Based on docking scores, compounds Undec-10-ynoic acid (C31039), Cyclohexanepropanoic acid (C69702), Hexanoic acid, cyclohexyl ester (C80388), Octanal, 2-(phenylmethylene)-(C1550884), Nerolidol (C5284507), and Octanal, 2-(phenylmethylene)-(Cinnamaldehyde, α-hexyl-) (C10502404) were identified as promising competitors of the dichromate ion for both receptors.

### 2.3. ADMET

An in silico ADMET analysis was conducted for all 49 phytochemical compounds using the OSIRIS Property Explorer (Idorsia Pharmaceuticals Ltd., Allschwil, Switzerland), providing insights into their absorption, distribution, metabolism, excretion, and toxicity profiles. Among the analyzed compounds, 18 were predicted to pose no risk of mutagenicity, tumorigenicity, irritancy, or reproductive toxicity (Table 3). Of these, 11 compounds exhibited favorable physicochemical properties, with calculated cLogP values ranging from 0.7 to 2.96, solubility (LogS) values between −1.51 and −3.74, and drug-score values spanning 0.42 to 0.82, indicating a promising balance of drug-likeness and safety for potential application.

Based on the combined docking and ADMET results, two compounds were selected for each receptor for further investigation due to their favorable binding affinities and acceptable pharmacokinetic and toxicity profiles. Detailed analysis of the binding modes within the sulfate transporter 7LHV and the phosphate transporter 7SP5 revealed that the studied compounds share several key interacting residues with both dichromate and the reference sulfate and phosphate complexes (Table 4 and Table 5).

Notably, residues Tyr116, Ala153, Phe391, Ser392, and Arg393 were consistently involved in ligand recognition and stabilization within 7LHV, indicating their central role in shaping the binding pocket architecture. Similarly, in 7SP5, residues Asn82, Tyr150, and Lys459 were repeatedly observed as major contributors to ligand binding, highlighting their importance in phosphate transport and interaction specificity. The recurrence of these residues across different ligands suggests that they act as hotspot amino acids critical for substrate recognition and competitive binding.

### 2.4. Molecular Dynamics

Based on docking scores, binding mode, and ADMET predictions, compound C69702 was identified as the most promising ligand for both 7LHV and 7SP5 receptors, exhibiting favorable binding affinities and ADMET properties. Its potential inhibitory activity is attributed to the establishment of key interactions with critical active-site residues in both targets. To further evaluate the stability and persistence of these interactions, molecular dynamics simulations were performed for each ligand–receptor complex. The systems were analyzed using key dynamic parameters, including RMSD, RMSF, and protein-ligand interaction profiles.

**Figure 1:**
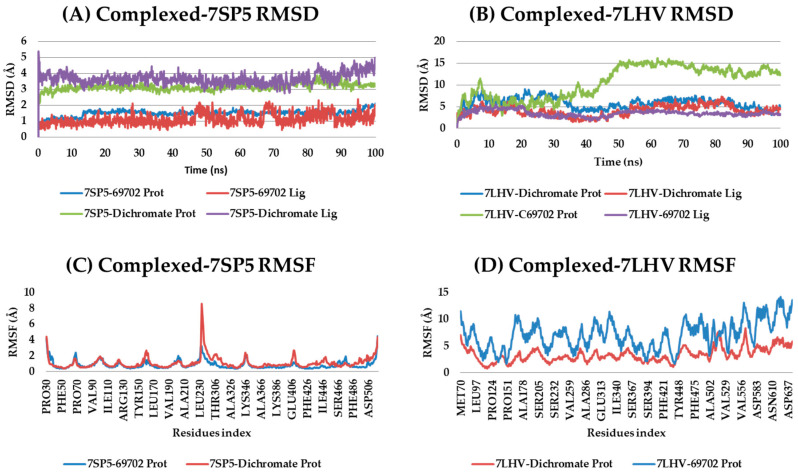
(**A**,**B**) RMSD and (**C**,**D**) RMSF plots of the complexes formed by C69702 and dichromate with the 7SP5 and 7LHV receptors during molecular dynamics simulations.

RMSD analysis revealed distinct dynamic behaviors for the 7SP5 complexes during the 100 ns simulation (Figure 1A,B). The protein in the 7SP5–C69702 complex maintained lower structural deviations (~1–2 Å) compared with the 7SP5–dichromate complex (~2.5–3.5 Å), indicating greater conformational stability in the presence of C69702. Similarly, ligand RMSD analysis showed that C69702 remained more stable within the binding pocket, fluctuating around 0.5–2 Å, whereas dichromate exhibited higher deviations (3–5 Å). These results suggest that C69702 forms a more stable and favorable complex with the 7SP5 receptor compared with dichromate.

For the 7LHV complexes, the protein in the 7LHV–dichromate system exhibited moderate fluctuations between 3 and 7 Å, indicating better overall structural stability, whereas the protein in the 7LHV–C69702 complex showed a marked increase after 45 ns, reaching approximately 15 Å, suggesting significant conformational rearrangements. In contrast, both ligands remained relatively stable during the simulation, with dichromate fluctuating between 2 and 5 Å and C69702 maintaining lower deviations below 4 Å, confirming stable accommodation within the binding pocket.

The root mean square fluctuation profiles were analyzed to evaluate residue-level flexibility of the protein in different ligand-bound states (C69702 and dichromate) for both 7LHV and 7SP5 systems (Figure 1C,D). For 7SP5, the C69702 complex exhibited overall lower fluctuation amplitudes compared with the dichromate complex, indicating reduced local flexibility and suggesting that C69702 contributes to better stabilization of the receptor structure during the simulation. In contrast, the higher fluctuations observed in the dichromate complex reflect greater residue mobility and comparatively lower structural stability.

For 7LHV, both complexes displayed comparable fluctuation profiles across most residues, although the C69702 complex showed slightly higher residue mobility in several regions, consistent with the elevated protein RMSD values observed during the simulation. These findings indicate that while C69702 promotes greater stability in the 7SP5 system, the dichromate complex maintains comparatively better structural stability in the 7LHV receptor.

**Figure 2:**
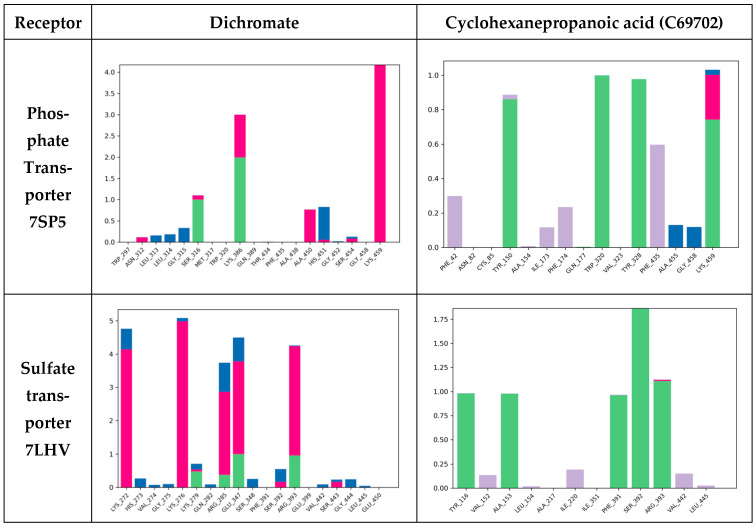
Protein–ligand contact histograms of the simulated complexes formed by dichromate and C69702 with the 7SP5 and 7LHV receptors during molecular dynamics simulations. Interaction types are represented as follows: pink, ionic bonds; violet, hydrophobic interactions; green, hydrogen bonds; blue, water bridges.

The analysis of the ligand–protein contact histograms for the 7SP5 receptor identified a limited number of key residues responsible for stable ligand binding (Figure 2). For the dichromate complex, the dominant interactions are primarily electrostatic, characterized by persistent ionic contacts with Lys386 and Lys459, along with a hydrogen bond involving Ser316. These residues represent the main anchoring points of the ligand within the binding site, suggesting that dichromate binding is predominantly governed by charge–charge interactions.

In contrast, the C69702 ligand exhibits high-frequency interactions with Tyr150, Trp320, Tyr328, and Lys459 through hydrogen bonding, together with an ionic interaction with Lys459 and hydrophobic contacts involving Phe42, Phe174, and Phe435. This combination of polar and nonpolar interactions indicates a more balanced and complementary binding mode, promoting improved ligand accommodation and enhanced stability within the binding pocket.

Focusing exclusively on these high-occupancy contacts, the results reveal a clear distinction between the two ligands. Dichromate relies on a restricted set of strong ionic interactions, whereas C69702 engages a broader interaction network involving both hydrogen bonding and hydrophobic stabilization. This difference in interaction patterns is consistent with the dynamic behavior observed in molecular dynamics simulations, where the C69702 complex displays reduced local flexibility compared to the dichromate-bound system.

The interaction profiles of dichromate and compound C69702 with the 7LHV receptor reveal distinct binding patterns. Dichromate exhibits a network of hydrogen and ionic interactions, which contribute to complex stabilization within the binding pocket. For Dichromate, the binding mode is primarily stabilized through multiple ionic interactions involving key positively charged residues, including Lys272, Lys276, Arg285, Arg393, and Glu347. These electrostatic contacts suggest a strong anchoring effect of dichromate within the active site, particularly through interactions with lysine and arginine residues, which are commonly involved in ligand stabilization due to their charged side chains.

In contrast, compound C69702 exhibits a distinct interaction profile. It forms hydrogen bonds with Tyr116, Ala153, Phe391, Ser392, and Arg393, which contribute to the stabilization of the ligand within the binding pocket. This hydrogen-bonding network differs from that observed with dichromate, highlighting a more specific and directed binding mode.

The two ligands share only one common interacting residue, Arg393, highlighting its central role in ligand recognition and stabilization within the binding pocket. C69702 establishes additional hydrogen bonds with several active-site residues, indicating a more extensive interaction network and a potentially more specific binding mode. However, RMSD analysis of the 7LHV–C69702 complex reveals an unstable trajectory characterized by significant structural fluctuations throughout the simulation. Overall, although C69702 shows a higher number of protein contacts, its dynamic instability suggests a less stable binding behavior compared to a more conformationally stable ligand–receptor complex.

The compound cyclohexanepropanoic acid (C69702), identified from *Aloe djiboutiensis*, emerged as the most promising competitive agent against dichromate for both sulfate and phosphate transporters. This compound demonstrated favorable binding energies and an advantageous binding mode, together with acceptable ADMET properties, indicating its potential as a safe and effective inhibitor. Furthermore, molecular dynamics analyses revealed the strength and persistence of its interactions with key active-site residues, as well as the overall stability of the formed complexes throughout the simulation period. These findings suggest that cyclohexanepropanoic acid may serve as a potential candidate for limiting dichromate transport through competitive inhibition mechanisms.

The present findings provide valuable insights into the volatile phytochemical composition of ten Djiboutian plant species, a flora that remains relatively underexplored. Although several of the identified compounds have previously been reported in other plant species, their occurrence in the investigated plants contributes to the phytochemical characterization of Djiboutian biodiversity. Furthermore, the identified volatile constituents served as a basis for the in silico screening, which highlighted several compounds with favorable interactions toward sulfate and phosphate transporters implicated in dichromate uptake. These results suggest that naturally occurring phytochemicals may represent potential candidates for modulating transporter-mediated chromium accumulation in plants. Nevertheless, further phytochemical investigations employing complementary analytical techniques and experimental validation studies are required to confirm the biological relevance of the predicted interactions.

## 3. Materials and Methods

### 3.1. GC–MS Analysis of Plant Extracts

The phytochemical composition of the studied plant extracts was investigated using gas chromatography–mass spectrometry (GC–MS) [12]. The aerial parts and leaves of ten Djiboutian medicinal plants were collected, dried at room temperature, and ground into fine powder prior to extraction. The extraction yields obtained for the different plant materials ranged from 2% to 16%, depending on the species and the plant part used.

The prepared extracts were analyzed using a GC–MS system equipped with a capillary column suitable for volatile and semi-volatile compound separation. Helium was used as the carrier gas under constant flow conditions. The injector and detector temperatures were maintained under optimized analytical conditions, while the oven temperature program was adjusted progressively to ensure efficient separation of the phytochemical constituents.

Compound identification was performed by comparing the obtained mass spectra with those available in the NIST mass spectral library and by considering retention times and fragmentation patterns reported in the literature [12,13]. Relative percentages of the detected compounds were calculated from peak area normalization without correction factors.

The GC–MS investigation enabled the identification of 49 phytochemical constituents distributed among the ten medicinal plants. The identified metabolites belonged to various chemical classes, including alcohols, terpenoids, hydrocarbons, ketones, aldehydes, esters, nitrogen-containing compounds, phenolics, and fatty acid derivatives. These identified compounds constituted the ligand library subsequently used for molecular docking, ADMET prediction, and molecular dynamics analyses.

### 3.2. Molecular Docking’s Material

#### 3.2.1. Ligand Dataset Preparation

Ten Djiboutian medicinal plants, including *Boscia coriacea* (M1), *Maerua triphylla* (M2), *Becium filamentosum* (M3), *Jasminum floribundum* (M4), *Heliotropium longiflorum* (M5), *Caesalpinia erianthera* (M6), *Cadaba rotundifolia* (M7), *Aloe djiboutiensis* (M8), *Aloe ericahenriettae* (M9), and *Pulicaria somalensis* (M10), were previously investigated for their phytochemical composition. This analysis led to the identification of 49 unique phytoconstituents after the removal of duplicate compounds. The chemical structures of all identified metabolites were retrieved from the PubChem database and subjected to geometry optimization to obtain energetically stable conformations prior to molecular docking analysis [14]. To ensure accurate representation of the target toxicant, the dichromate ion (Cr_2_O_7_^2−^) was manually constructed and optimized while preserving its charged state and structural geometry. In addition, sulfate (SO_4_^2−^) and phosphate (PO_4_^3−^) ions were included as reference ligands to provide comparative insight into transporter selectivity and binding behavior. Ligand preparation was carried out by the addition of Gasteiger partial charges, followed by the assignment of rotatable bonds for flexible compounds where applicable, ensuring appropriate conformational sampling during docking simulations.

#### 3.2.2. Protein Preparation

The sulfate transporter AtSULTR4;1 from Arabidopsis thaliana (PDB ID: 7LHV; 2.75 Å), co-crystallized with sulfate, and the eukaryotic phosphate transporter (PDB ID: 7SP5; 2.9 Å), co-crystallized with phosphate, were selected due to their high structural quality and direct biological relevance to sulfate- and phosphate-mediated transport processes. The presence of native co-crystallized ligands provides experimentally validated binding sites, allowing a reliable evaluation of ligand–transporter interactions. Moreover, sulfate and phosphate transporters are recognized pathways for chromate and dichromate uptake because of the structural similarity between these oxyanions, making 7LHV and 7SP5 appropriate targets for investigating the potential of phytochemicals to interfere with dichromate transport in plants. The three-dimensional structures of the sulfate transporter (PDB ID: 7LHV sulfate transporter structure) [3] and the phosphate transporter (PDB ID: 7SP5 phosphate transporter structure) [4] were retrieved from the Protein Data Bank. Both protein structures were subjected to energy minimization using Swiss-PdbViewer v.4.1 to optimize geometry and eliminate steric clashes [15]. Preprocessing steps included the removal of crystallographic water molecules and non-essential heteroatoms, followed by the addition of polar hydrogen atoms and the assignment of Gasteiger partial charges using AutoDock Tools v.4.2.6 [8].

#### 3.2.3. Molecular Docking Protocol

Molecular docking simulations were performed using AutoDock 4 [8], selected for its ability to handle charged ligands and metal-containing species such as dichromate [5]. All phytochemical compounds, along with dichromate and the reference ligands (sulfate and phosphate), were docked into the active sites of both transporters to evaluate their binding affinities and interaction modes [6].

For each receptor, the docking grid box was centered on the position of the co-crystallized ligand (sulfate for the sulfate transporter and phosphate for the phosphate transporter), ensuring accurate targeting of the substrate-binding pockets. Docking calculations were carried out using the Lamarckian Genetic Algorithm (LGA) with multiple independent runs to ensure sufficient conformational sampling and reproducibility. For each docked complex, the binding energy (ΔG, expressed in kcal/mol) and the inhibition constant (Ki) were calculated, providing quantitative measures of ligand affinity and predicted inhibitory potential.

#### 3.2.4. Validation and Interaction Analysis

The docking protocol was validated by re-docking the native ligands (sulfate and phosphate) into their respective binding sites and comparing the predicted poses with their crystallographic conformations. The best-ranked conformations were selected based on the lowest binding energy values and corresponding inhibition constant Ki. Protein–ligand interactions, including hydrogen bonding, electrostatic interactions, and hydrophobic contacts, were analyzed using Discovery Studio Visualizer 2023 [16]. Special attention was given to identifying key residues involved in ligand recognition and binding stability in both transporters.

### 3.3. ADMET and Drug-Likeness Prediction

An in silico ADMET analysis was conducted for all 49 phytochemical compounds using the OSiRIS Property Explorer (Idorsia Pharmaceuticals Ltd., Allschwil, Switzerland) [17]. Parameters evaluated included mutagenicity, tumorigenicity, irritancy, and reproductive toxicity, along with physicochemical descriptors such as molecular weight (MW), cLogP, solubility (LogS), and drug score [18]. Compounds with favorable toxicity profiles and acceptable drug-like properties were prioritized for further docking analysis [9].

### 3.4. Comparative Analysis Strategy

A comparative analysis was performed between the sulfate transporter 7LHV and the phosphate transporter 7SP5 to evaluate differences in ligand binding affinities, interaction patterns, and competitive inhibition potential. This dual-target approach enabled a comprehensive assessment of the ability of plant-derived compounds to interfere with anion transport pathways involved in dichromate uptake.

### 3.5. Molecular Dynamics Simulations

Molecular dynamics (MD) simulations were performed on the most promising ligand–receptor complexes selected based on docking scores and ADMET properties. All simulations were carried out using the Schrödinger software suite, where the Maestro (Version 12.5.139, Release 2020-3, Schrödinger, LLC, New York, NY, USA, 2020) was employed for system preparation, geometry optimization, energy minimization, solvation, and equilibration steps [19]. The production MD simulations were executed using Desmond under physiological conditions [20]. Each complex was subjected to a 100 ns production MD simulation to ensure adequate sampling of conformational space and reliable evaluation of system stability.

Key structural and dynamic parameters, including root mean square deviation (RMSD), root mean square fluctuation (RMSF), and protein–ligand interaction profiles, were computed and analyzed throughout the simulation trajectories. These analyses were used to assess the structural stability of the complexes and to characterize the strength, persistence, and nature of the intermolecular interactions over time [11].

## 4. Conclusions

The present study demonstrated the effectiveness of an integrated in silico approach combining GC–MS phytochemical profiling, molecular docking, ADMET prediction, and molecular dynamics simulations to identify potential natural inhibitors of dichromate uptake in plants. A total of 49 phytochemical compounds identified from ten Djiboutian medicinal plants were screened against the sulfate transporter 7LHV and the phosphate transporter 7SP5, revealing several metabolites with promising binding affinities and favorable interaction profiles.

Comparative docking analyses showed that multiple phytochemicals were able to interact with key amino acid residues involved in sulfate and phosphate recognition, suggesting their potential to competitively interfere with dichromate transport pathways. Among the investigated compounds, cyclohexanepropanoic acid identified from *Aloe djiboutiensis* exhibited the most promising inhibitory profile, combining strong binding affinity, acceptable ADMET characteristics, and stable intermolecular interactions during molecular dynamics simulations. The RMSD, RMSF, and protein–ligand contact analyses confirmed the structural stability of the formed complexes and highlighted the importance of key residues involved in ligand stabilization.

Overall, the obtained findings suggest that phytochemicals derived from Djiboutian medicinal plants may constitute promising eco-friendly candidates for limiting Cr(VI) uptake through competitive inhibition of sulfate and phosphate transporters. This study provides a theoretical foundation for future experimental investigations aimed at validating the inhibitory activity of these compounds under biological and environmental conditions. Furthermore, the proposed strategy may contribute to the development of sustainable approaches for reducing chromium accumulation in plants and improving agricultural and environmental safety in contaminated ecosystems.

## Figures and Tables

**Table 1:** Studied plants, plant parts used for extraction, extract masses, and extraction yields.

Code	Plant Name	Plant Part Used	Extract Mass (g)	Yield (%)
**M1**	*Boscia coriacea (Pax.)*	Leaves	5.44	11
**M2**	*Maerua triphylla (Thunb.)*	Leaves	5.18	10
**M3**	*Becium filamentosum (Forssk.)*	Leaves	6.03	12
**M4**	*Jasminum. floribundum (R.Br. ex Fresen.)*	Leaves	8.21	16
**M5**	*Heliotropium longiflorum (A.DC.)*	Leaves + Flowers	3.92	8
**M6**	*Caesalpinia erianthera*	Leaves	3.00	6
**M7**	*Cadaba rotundifolia*	Leaves	0.50	3
**M8**	*Aloe djiboutiensis*	Leaves	0.2	5
**M9**	*Aloe ericahenriettae*	Leaves	0.10	2
**M10**	*Pulcaria somalensis*	Leaves	5.10	10

**Table 2:** Binding energy (BE) and inhibition constant (Ki) values of the docked compounds compared with those of the dichromate, sulfate, and phosphate ions.

ID	Compounds	7lhv		7sp5	
		Binding Energy	Inhibition Constant	Binding Energy	Inhibition Constant
		Kcal/mol	Ki	Kcal/mol	Ki
**--**	**Dichromate**	−9.59	92.64 nM	−10.13	37.58 nM
**--**	**Sulfate**	−3.77	1.71 mM	−2.13	27.5 mM
**2969**	**n-Decanoic acid**	−5.87	49.44 µM	−5.93	44.85 µM
**3327**	**2,6,10-Dodecatrien-1-ol, 3,7,11-trimethyl**	−5.48	96.34 µM	−6.19	28.98 µM
**6548**	**3-Octanol, 3,7-dimethyl-**	−5.37	115.38 µM	−5.29	133.14 µM
**7211**	**1,3-Hexanediol, 2-ethyl-**	−4.54	469.98 µM	−4.74	336.88 µM
**7555**	**Triallyl cyanurate**	−6.27	25.17 µM	−5.76	60.09 µM
**7991**	**Pentanoic acid**	−4.46	542.42 µM	−4.29	717.36 µM
**8146**	**Ethanol, 2-(2-ethoxyethoxy)-**	−3.33	3.6 mM	−2.97	6.61 mM
**9461**	**1,1′-Biphenyl, 4-fluoro-**	−5.83	53.36 µM	−5.71	65.37 µM
**10281**	**Thymoquinone**	−5.85	51.65 µM	−5.13	174.2 µM
**10400**	**Cycloheptanone**	−4.59	431.1 µM	−4.75	332.22 µM
**10943**	**Benzene, 1,3-dichloro-**	−4.59	435.53 µM	−4.39	602.2 µM
**11169**	**Cyclotetrasiloxane, octamethyl-**	−3.38	3.33 mM	-	-
**11704**	**Stannane, tetraethyl-**	−5.82	54.05 µM	−5.91	46.58 µM
**12408**	**Octacosane**	−3.11	5.27 mM	-	-
**15969**	**Swep**	−5.90	46.99 µM	−5.15	168.7 µM
**17812**	**11-Bromoundecanoic acid**	−5.80	55.88 µM	−4.97	228.26 µM
**18840**	**Cyclopentylcarboxylic acid**	−5.36	116.97 µM	−4.79	309.01 µM
**26955**	**3-Buten-2-one, 4-(2,6,6-trimethyl-1-cyclohexen-1-yl)-**	−6.05	36.54 µM	−5.98	41.04 µM
**30875**	**1,13-Tetradecadiene**	−5.25	141.75 µM	−4.58	439.81 µM
**31039**	**Undec-10-ynoic acid**	−6.59	14.85 µM	−6.10	39.45 µM
**31278**	**Octadecanoic acid, butyl ester**	−4.42	577.6 µM	−2.68	10.79 mM
**61027**	**10-Undecenoic acid, butyl ester**	−5.03	206.99 µM	−5.28	135.0 µM
**69460**	**Quinoline, 1,2,3,4-tetrahydro-**	−5.18	160.19 µM	−4.71	354.08 µM
**69702**	**Cyclohexanepropanoic acid**	−6.62	14.1 µM	−6.40	20.52 µM
**74400**	**1,9-Decadiyne**	−5.18	160.12 µM	−4.71	353.88 µM
**77196**	**Cyclooctanemethanol**	−4.99	221.36 µM	−5.10	183.91 µM
**77986**	**1,1′-Biphenyl, 4-phenoxy-**	−6.36	21.79 µM	−0.62	353.51 mM
**80388**	**Hexanoic acid, cyclohexyl ester**	−6.65	13.42 µM	−6.22	27.66 µM
**85651**	**Hexadecanoic acid, octyl ester**	−4.26	753.31 µM	-	-
**87789**	**3-Hexadecanone**	−5.06	196.18 µM	−5.21	152.97 µM
**92798**	**Cyclopentaneethanamine, N,à-dimethyl**	−5.79	56.74 µM	−5.87	50.17 µM
**98972**	**4-Tridecanol**	−5.40	109.97 µM	−5.65	72.66 µM
**107378**	**2,2,4-Trimethyl-3-pentanol**	−4.70	361.65 µM	−4.79	307.34 µM
**123437**	**3-Nonyn-1-ol**	−4.37	628.28 µM	−4.05	1.07 mM
**136748**	**Isothiazole, 4-phenyl-**	−5.68	68.77 µM	−5.53	88.89 µM
**137138**	**3-Tetradecanol**	−4.99	219 µM	−4.66	386.71 µM
**138971**	**6-Methyl-1,5-heptadiene**	−4.05	1.08 mM	−4.18	857.18 µM
**140477**	**Hexadecanedioic acid, dimethyl ester**	−4.96	223.29 µM	−4.92	246.67 µM
**144415**	**Citric acid, tripentyl ester**	−2.91	7.3 mM	-	-
**219794**	**5-Hydroxy-4-octanone**	−4.99	220.23 µM	−4.71	352.28 µM
**445639**	**Oleic Acid**	−7.04	6.91 µM	−3.76	1.76 mM
**637563**	**Anethole**	−5.31	127.46 µM	−4.78	315.4 µM
**638072**	**Supraene**	−6.48	17.86 µM	-	-
**1550884**	**Octanal, 2-(phenylmethylene)-**	−6.25	26.1 µM	−6.50	15.71 µM
**5284507**	**Nerolidol**	−6.38	21.0 µM	−6.26	25.89 µM
**5354342**	**Oleic acid, butyl ester**	−4.37	629.21 µM	-	-
**5362810**	**4-Methyl-1,4-heptadiene**	−4.07	1.04 mM	−4.17	877.38 µM
**10502404**	**Octanal, 2-(phenylmethylene)- (Cinnamaldehyde, α-hexyl-)**	−6.43	19.33 µM	−6.16	30.7 µM
**10504339**	**Oleic acid (9-Octadecenoic acid, Z-)**	−6.71	12.05 µM	−2.82	8.57 mM

**Table 3:** Predicted ADMET properties of the studied compounds, highlighting their toxicity risk profiles (red: high risk; orange: moderate risk; green: no risk).

Id	Mutagenic	Tumorigenic	Irritant	Reproductive Effect	cLogP	Solubility	MW	TPSA	Drug Likeness	Drug-Score
**C92798**	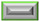	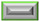	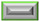	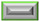	1.82	−2.17	141	12.03	1.04	0.82
**C136748**	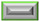	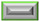	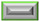	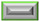	2.59	−1.94	161	41.13	0.81	0.79
**C69460**	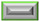	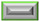	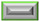	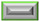	1.68	−2.29	133	12.03	0.65	0.78
**C219794**	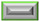	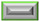	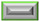	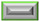	1.63	−1.86	144	37.3	−0.6	0.65
**C18840**	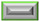	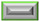	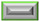	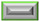	0.7	−1.51	114	37.3	−4.07	0.5
**C10400**	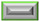	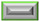	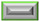	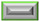	1.69	−2.07	112	17.07	−9.03	0.48
**C69702**	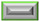	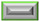	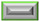	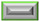	1.95	−2.33	156	37.3	−5.94	0.47
**C77196**	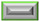	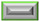	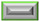	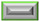	2.93	−2.34	142	20.23	−9.8	0.45
**C31039**	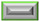	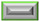	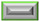	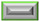	2.96	−2.94	182	37.3	−15.58	0.44
**C74400**	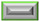	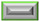	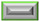	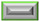	2.95	−3.25	134	0	−1.42	0.43
**C15969**	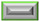	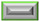	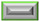	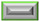	2.78	−3.74	219	38.33	−4.85	0.42
**C6548**	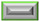	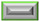	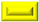	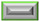	3.28	−2.47	158	20.23	−1.88	0.4
**C5362810**	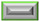	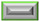	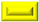	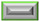	3.42	−2.17	110	0	−5.77	0.36
**C10281**	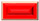	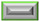	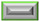	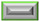	1.64	−1.68	164	34.14	−1.2	0.35
**C98972**	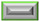	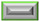	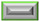	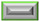	4.95	−3.55	200	20.23	−17.8	0.34
**C3327**	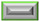	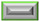	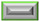	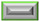	5.66	−2.86	222	20.23	−3.38	0.32
**C7211**	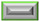	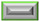	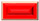	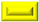	1.52	−1.59	146	40.46	−1.04	0.29
**C7991**	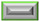	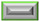	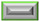	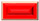	1.06	−1.27	102	37.3	−7.06	0.29
**C107378**	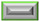	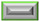	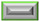	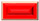	2.32	−1.89	130	20.23	−4.84	0.28
**C140477**	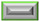	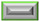	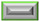	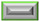	5.64	−3.97	314	52.6	−22.84	0.28
**C77986**	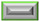	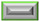	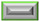	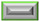	4.71	−6	246	9.23	−1.96	0.27
**C138971**	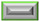	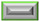	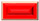	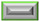	3.42	−2.17	110	0	−8.92	0.27
**C9461**	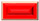	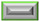	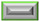	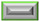	3.42	−4.02	172	0	−2.06	0.26
**C123437**	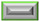	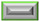	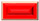	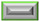	2.86	−4.01	140	20.23	−18	0.24
**C10502404**	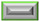	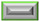	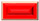	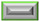	4.49	−3.71	216	17.07	−19.82	0.21
**C1550884**	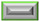	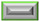	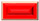	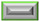	4.49	−3.71	216	17.07	−19.82	0.21
**C144415**	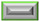	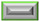	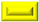	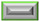	4.46	−3.64	402	99.13	−9.32	0.21
**C5284507**	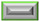	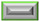	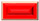	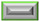	5.4	−3.12	222	20.23	−6.38	0.19
**C137138**	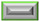	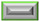	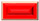	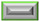	5.41	−3.82	214	20.23	−19.73	0.18
**C61027**	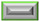	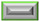	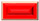	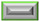	5.35	−3.8	240	26.3	−25.8	0.18
**C10943**	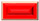	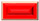	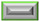	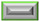	2.87	−3.09	146	0	−2.18	0.17
**C30875**	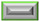	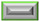	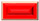	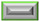	6.06	−4.1	194	0	−15.02	0.16
**C2969**	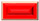	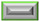	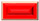	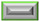	3.34	−2.62	172	37.3	−25.22	0.16
**C80388**	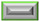	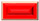	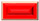	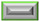	3.5	−3.24	198	26.3	−18.4	0.15
**C87789**	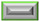	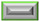	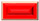	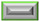	6.29	−4.42	240	17.07	−20.88	0.15
**C7555**	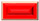	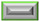	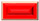	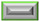	2.9	−3.38	249	66.36	−7.58	0.15
**C638072**	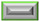	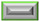	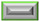	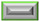	13.1	−6.3	410	0	−3.52	0.14
**C85651**	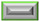	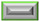	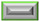	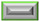	9.62	−6.29	368	26.3	−30.2	0.14
**C12408**	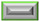	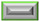	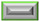	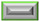	12.79	−8	394	0	−20.4	0.12
**C8146**	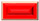	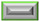	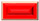	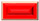	−0.13	−0.32	134	38.69	−21.68	0.11
**C5354342**	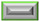	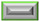	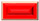	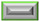	8.46	−5.52	338	26.3	−33	0.1
**C637563**	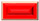	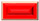	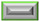	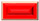	2.68	−2.54	148	9.23	−3.42	0.1
**C31278**	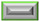	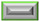	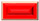	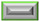	8.71	−5.75	340	26.3	−29.23	0.09
**C26955**	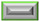	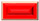	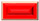	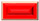	3.32	−2.74	192	17.07	−6.41	0.09
**C11704**	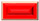	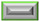	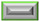	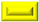	3.79	−10.45	236	0	−4.94	0.08
**C11169**	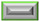	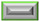	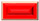	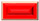	5.57	−7.78	296	36.92	−68	0.06
**C10504339**	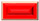	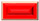	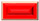	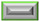	6.72	−4.55	282	37.3	−28.97	0.05
**C445639**	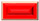	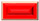	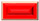	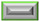	6.72	−4.55	282	37.3	−28.97	0.05
**C17812**	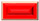	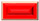	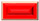	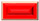	4.15	−3.43	264	37.3	−33.26	0.05

**Table 4:** Comparison of the two best-selected compounds, dichromate ion and sulfate ion, against the 7LHV receptor based on binding energy (BE), inhibition constant (Ki), and drug score, together with their corresponding 2D interaction visualizations.

7LHV	Sulfate	Dichromate		C31039		C69702	
BE kcal/mol	−3.77	−9.59		−6.59		−6.62	
Ki	1.71 mM	92.64 nMol		14.85 µM		14.1 μMol	
DS	-	-		0.44		0.47	
2D	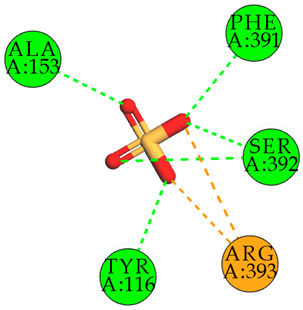	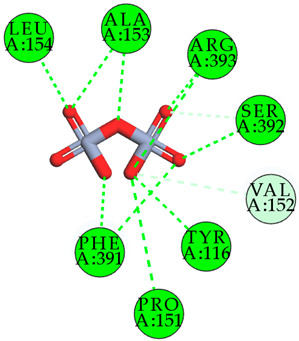		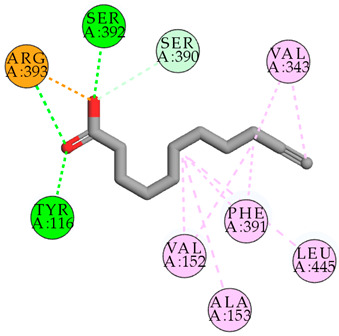		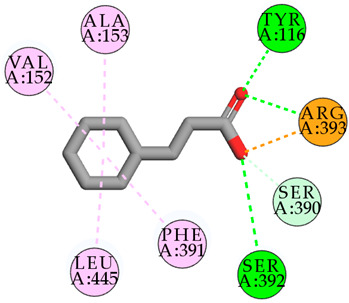	
Residues	Tyr116 Ala153 Phe391 Ser392 Arg393	Tyr116 Pro151 Val152 Ala153	Leu154 Phe391 Ser392 Arg393	Tyr116 Val152 Ala153 Val343 Ser390	Phe391 Ser392 Arg393 Leu445	Tyr116 Val152 Ala153 Ser390 Phe391	Ser392 Arg393 Leu445

**Table 5:** Comparison of the two best-selected compounds, dichromate ion and sulfate ion, against the 7SP5 and 7LHV receptors based on binding energy (BE), inhibition constant (Ki), and drug score, together with their corresponding 2D interaction visualizations.

7SP5	Phosphate	Dichromate	C31039	C69702
BE kcal/mol	−2.13	−10.13	−6.01	−6.4
Ki	27.5 mM	37.58 nM	39.45 µM	20.52 µM
DS	-	-	0.44	0.47
2D	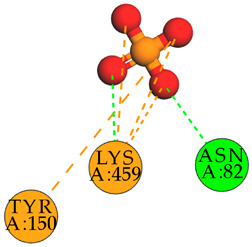	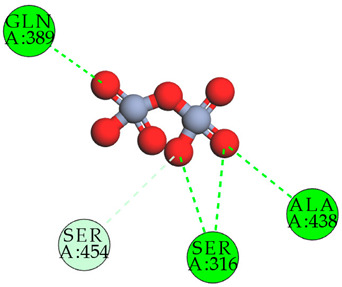	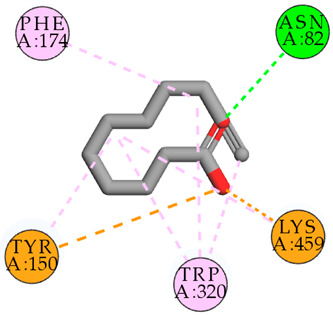	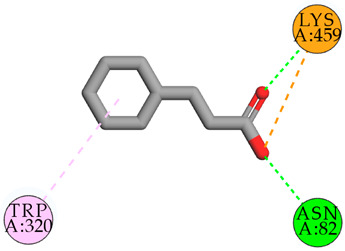
Residues	Asn82 Tyr150 Lys459	Ser316 Gln389 Ala438 Ser454	Asn82 Tyr150 Phe174 Trp320 Lys459	Asn82 Trp320 Lys459

## Data Availability

Data is contained within the article.

## Supplementary Materials

The following supporting information can be downloaded at: https://www.mdpi.com/article/10.3390/ph19071000/s1, Table S1: GC–MS identified compounds in Boscia coriacea (M1) extract.; Table S2: GC–MS identified compounds in Maerua triphylla (M2) extract; Table S3: GC–MS identified compounds in Becium filamentosum (M3) extract; Table S4: GC–MS identified compounds in Jasminum floribundum (M4) extract; Table S5: GC–MS identified compounds in Heliotropium longiflorum (M5) extract; Table S6: GC–MS identified compounds in Caesalpinia erianthera (M6) extract; Table S7: GC–MS identified compounds in Cadaba rotundifolia (M7) extract; Table S8: GC–MS identified compounds in Aloe djiboutiensis (M8) extract; Table S9: GC–MS identified compounds in Aloe ericahenriettae (M9) extract; Table S10: GC–MS identified compounds in Pulicaria somalensis (M10) extract.
