# Supplementary material for: In Silico Investigation of Phytochemicals from Djiboutian Plants Targeting Sulfate and Phosphate Transporters Involved in Dichromate Uptake
Source: Pharmaceuticals (Basel). 2026 Jun 28;19(7):1000. doi: 10.3390/ph19071000 (PMC13414512; doi:10.3390/ph19071000)
Supplement: Supplementary file 1 [file pharmaceuticals-19-01000-s001.zip › SM.pdf]

In silico investigation of phytochemical inhibitors targeting sulfate and phosphate transporters to mitigate dichromate uptake in Djiboutian plants

Supplementary Material

GC–MS phytochemical profiling of the investigated medicinal plants

Table S1. GC–MS identified compounds in *Boscia coriacea* (M1) extract.

Retention time (min)	Compound name	Area (%)
6.04	Docosanoic acid (n-Docosanoic acid)	3.20
6.13	Disiloxane, hexamethyl- (Bis(trimethylsilyl) ether)	2.30
8.35	Disiloxane, hexamethyl- (Bis(trimethylsilyl) ether)	19.64
46.23	Eicosanoic acid (Arachidic acid)	8.07
51.39	Oleic acid (9-Octadecenoic acid, Z-)	1.45
51.63	Octanal, 2-(phenylmethylene)- (Cinnamaldehyde, α-hexyl-)	7.29
51.93	Cycloheptanone	3.79
52.81	Octanal, 2-(phenylmethylene)- (Cinnamaldehyde, α-hexyl-)	2.81
65.82	1,3-Hexanediol, 2-ethyl-	1.49
71.04	Supraene	4.26
76.87	Eicosanoic acid	11.42
80.03	2-Bromotetradecanoic acid	5.89
80.33	à-Endosulfan	1.72
80.81	2-Bromotetradecanoic acid	25.13
81.52	Heptachlor epoxide	1.53
		**99.99**

Table S2. GC–MS identified compounds in *Maerua triphylla* (M2) extract.

Retention time (min)	Compound name	Area (%)
8.35	Disiloxane, hexamethyl-	8.91
22.56	1,1'-Biphenyl, 4-fluoro-	10.34
34.15	2,4,5-T Methyl ester	4.3
40.54	1,1'-Biphenyl, 4-phenoxy-	3.09
42.25	Benzene, 1,4-dichloro-2-nitro-	1.87
42.36	Benzene, 1,2,4-tris-trimethylsilyl-	12.4
44.15	Phenol, 4,4'-thiobis-	16.53
46.23	Eicosanoic acid	2.79
51.63	Oleic Acid	3.69
72.30	Silane, tetramethyl-	2.32
73.09	Hexadecanoic acid, octyl ester	23.58
75.12	Diazepam	2.71
80.02	Heptachlor epoxide	1.75
		**94.28**

Table S3. GC–MS identified compounds in *Becium filamentosum* (M3) extract.

Retention time (min)	Compound name	Area (%)
6.05	Oleic acid, butyl ester	4.06
8.35	Disiloxane, hexamethyl-	21.64
23.76	3-Octanol, 3,7-dimethyl-	8.12
46.23	2-Bromotetradecanoic acid	10.42
51.94	Oleic Acid	5.16
52.80	Octanal, 2-(phenylmethylene)-	4.85
72.48	3-Tetradecanol	3
76.85	Dieldrin	3.62
79.89	2-Bromotetradecanoic acid	3.33
80.01	Oleic Acid	8.56
80.82	Endrin	3.46
81.21	4,7-Methano-1H-indene, 1,2,3,4,5,6,7,8,8-nonachloro-2,3,3a ,4,7,7a-hexahydro	3.49
81.40	10-Undecenoic acid, butyl ester	3.17
81.52	Bifenox	358
		**86.46**

Table S4. GC–MS identified compounds in *Jasminum floribundum* (M4) extract.

Retention time (min)	Compound name	Area (%)
8.35	Disiloxane, hexamethyl-	3.8
36.79	Cyclotetrasiloxane, octamethyl-	4.79
46.22	Hexanoic acid, cyclohexyl ester	10.9
51.61	3-Nonyn-1-ol	11.6
51.95	Oleic acid, butyl ester	4.71
52.78	Bifenox	4.05
66.71	Heptachlor epoxide	3.44
68.29	1,13-Tetradecadiene	3.52
68.91	Isopropalin	8.98
71.87	Cyclotetrasiloxane, octamethyl-	17.22
72.32	Isopropalin	12.73
76.02	3-Hexadecanone	3.3
80.00	Octadecanoic acid, butyl ester	3.93
81.09	Cyclopentaneethanamine, N,à-dimethyl	3.68
81.80	Endrin	3.36
		**100.01**

Table S5. GC–MS identified compounds in *Heliotropium longiflorum* (M5) extract.

Retention time (min)	Compound name	Area (%)
6.80	Benzene, 1-chloro-3-nitro-	1.64
6.86	Ethanol, 2-(2-ethoxyethoxy)-	1.98
8.11	Swep	1.44
8.34	Disiloxane, hexamethyl-	60.01
15.43	Disiloxane, hexamethyl-	6.88
46.22	2-Bromotetradecanoic acid	3.12
51.36	Undec-10-ynoic acid	2.44
51.60	Oleic acid, butyl ester	5.56
51.92	Triallyl cyanurate	2.08
52.78	Octanal, 2-(phenylmethylene)-	1.44
65.80	2-Thiophenecarboxaldehyde, 5-chloro	6.31
67.34	Cyclotetrasiloxane, octamethyl-	1.93
80.00	4-Methyl-1,4-heptadiene	1.51
81.26	Phosphorodithioic acid, O-(2,4-dichlorophenyl) O-ethyl S-propyl ester	1.62
81.62	Cyclopentane, bromo-	2.04
		**100**

Table S6. GC–MS identified compounds in *Caesalpinia erianthera* (M6) extract.

Retention time (min)	Compound name	Area (%)
8.34	Disiloxane, hexamethyl-	30.05
41.37	6-Methyl-1,5-heptadiene	3.73
46.21	n-Decanoic acid	8.98
50.16	3-Buten-2-one,4-(2,6,6-trimethyl-1-cyclohexen-1-yl)-	3.54
51.60	Oleic acid, butyl ester	8.38
51.93	á-Endosulfan	4.37
52.77	Docosanoic acid	4.29
57.26	Supraene	2.88
60.71	Tridiphane	5.96
63.62	Stannane, tetraethyl-	7.61
68.37	1-Chloroeicosane	3.44
73.63	Endrin	2.89
76.78	Oleic Acid	4.64
79.87	Oleic acid, butyl ester	3.21
79.99	Oleic Acid	6.01
		**99.98**

Table S7. GC–MS identified compounds in *Cadaba rotundifolia* (M7) extract.

Retention time (min)	Compound name	Area (%)
8.1	Methane, isothiocyanato	1.88
8.33	Disiloxane, hexamethyl-	38.88
15.43	Anethole	2.09
40.69	Phenol, 4,4'-thiobis-	3.92
40.93	Benzoyl chloride, 2,4-dichloro-	2.14
45.64	Anethole	2.42
46.21	Eicosanoic acid	9
51.59	Octanal, 2-(phenylmethylene)-	14.66
51.89	Endosulfan	1.81
52.76	Octanal, 2-(phenylmethylene)-	1.68
76.83	Dieldrin	3.34
79.1	2,2,4-Trimethyl-3-pentanol	2.39
79.99	2-Bromotetradecanoic acid	7.71
80.07	2,6,10-Dodecatrien-1-ol, 3,7,11-trimethyl	4.56
80.77	Supraene	3,51
		**99.99**

Table S8. GC–MS identified compounds in *Aloe djiboutiensis* (M8) extract.

Retention time (min)	Compound name	Area (%)
8.35	Disiloxane, hexamethyl-	49.98
15.35	Cyclotetrasiloxane, octamethyl-	2.75
36.69	Citric acid, tripentyl ester	1.86
46.21	Octanal, 2-(phenylmethylene)-	4.23
51.57	Oleic Acid	3.93
51.9	Cyclohexanepropanoic acid	2.08
52.77	Bifenox	2.13
65.29	1-(Nitromethyl)-cyclohexanol	1.64
76.23	Oleic acid, butyl ester	1.42
79.99	Isothiazole, 4-phenyl-	1.62
81.98	1-Bromo-2,4,6-triisopropylbenzene	1.6
82.25	1-Heptadecene	2.61
82.4	Octadecanoic acid, butyl ester	2.82
		**78.67**

Table S9. GC–MS identified compounds in *Aloe ericahenriettae* (M9) extract.

Retention time (min)	Compound name	Aire %
6.86	Benzene, 1,3-dichloro-	1.89
8.34	Disiloxane, hexamethyl-	48.2
15.34	Pentanoic acid	1.73
40.70	4-Tridecanol	1.56
51.33	á-Endosulfan	1.63
51.56	Oleic acid, butyl ester	5.83
51.91	Octadecanoic acid, butyl ester	3.56
52.74	Eicosanoic acid	2.75
75.72	Pendimethalin	1.6
75.98	Octacosane	4.02
79.98	Oleic Acid	6.7
81.05	Cyclooctanemethanol	1.51
81.57	Oleic acid, butyl ester	7.59
82.26	5-Hydroxy-4-octanone	4.6
		**93.17**

Table S10. GC–MS identified compounds in *Pulicaria somalensis* (M10) extract.

Retention time (min)	Compound name	Area (%)
8.34	Disiloxane, hexamethyl-	38.82
33.26	2-Tridecanone	2.46
33.88	Nerolidol	2.78
34.02	Adamantane, 1,3-dimethyl-	6.61
34.15	Tricyclazole	4.89
39.82	Thymoquinone	5.01
40.62	Bicyclo[6.1.0]nonane, 9,9-dibromo-,	2.15
46.20	Quinoline, 1,2,3,4-tetrahydro-	3.62
51.25	1,9-Decadiyne	10.44
51.57	Oleic Acid	2.76
52.75	Docosanoic acid	2
80.12	Hexadecanedioic acid, dimethyl ester	2.26
80.87	Cyclopentylcarboxylic acid	3.25
81.10	11-Bromoundecanoic acid	8.53
82.24	2,6,10-Dodecatrien-1-ol, 3,7,11-trimethyl	4.41
		**99.99**
